# Twofold processing for denoising ultrasound medical images

**DOI:** 10.1186/s40064-015-1566-6

**Published:** 2015-12-14

**Authors:** P. V. V. Kishore, K. V. V. Kumar, D. Anil kumar, M. V. D. Prasad, E. N. D. Goutham, R. Rahul, C. B. S. Vamsi Krishna, Y. Sandeep

**Affiliations:** Department of Electronics and Communications Engineering, K L University, Vaddeswaram, Guntur, India

**Keywords:** Ultrasound medical image denoising, Speckle noise, Block processing in wavelet domain, Hard and soft thresholding, Medical image fusion

## Abstract

Ultrasound medical (US) imaging non-invasively pictures inside of a human body for disease diagnostics. Speckle noise attacks ultrasound images degrading their visual quality. A twofold processing algorithm is proposed in this work to reduce this multiplicative speckle noise. First fold used block based thresholding, both hard (BHT) and soft (BST), on pixels in wavelet domain with 8, 16, 32 and 64 non-overlapping block sizes. This first fold process is a better denoising method for reducing speckle and also inducing object of interest blurring. The second fold process initiates to restore object boundaries and texture with adaptive wavelet fusion. The degraded object restoration in block thresholded US image is carried through wavelet coefficient fusion of object in original US mage and block thresholded US image. Fusion rules and wavelet decomposition levels are made adaptive for each block using gradient histograms with normalized differential mean (NDF) to introduce highest level of contrast between the denoised pixels and the object pixels in the resultant image. Thus the proposed twofold methods are named as adaptive NDF block fusion with hard and soft thresholding (ANBF-HT and ANBF-ST). The results indicate visual quality improvement to an interesting level with the proposed twofold processing, where the first fold removes noise and second fold restores object properties. Peak signal to noise ratio (PSNR), normalized cross correlation coefficient (NCC), edge strength (ES), image quality Index (IQI) and structural similarity index (SSIM), measure the quantitative quality of the twofold processing technique. Validation of the proposed method is done by comparing with anisotropic diffusion (AD), total variational filtering (TVF) and empirical mode decomposition (EMD) for enhancement of US images. The US images are provided by AMMA hospital radiology labs at Vijayawada, India.

## Background

Ultrasound medical images save many lives by early detection of fetus differences in pregnant woman for around two decades now. Compared with tomography (CT), magnetic resonance imaging (MRI) and Positron emission technology (PET), ultrasound (US) imaging is the safest for the sensitive fetus. Fetus scanning of pregnant woman enables gynecologists to check the health of the baby and mother satisfactorily. Ultrasound scanners come in handy to perform this job at lower costs and radiation effects. But the visual quality of images from ultrasound scanners is significantly poor because of speckle noise (Touzi 2002; Dinh-Hoan Trinh et al. 2014; Sonia et al. 2012; Huang and Xiaoping 2013). Lessening the effects of speckle at device and image level is extensively researched. Device level improvements in the form of 3D (Fenster and Downey 2013) and 4D ultrasound scanners (Solberg et al. 2011) are available. These improvements come at a cost that is unbearable by hospitals in poorer countries. Therefore, cheaper alternatives for improving visually as a necessary post processing step for 2D ultrasound images. These post processing steps include speckle noise removal, contrast enhancement and edge preserving methods. Speckle noise results from multiple reflections of ultrasound waves from the hard tissues of the scanned human body. The nature of speckle is multiplicative. Therefore difficult to model in real time, so inverse filtering methods to remove noise may be effective. In the last decade, there were denoising methods influenced by the fields of computer science, signal processing, probability and artificial intelligence (Compas et al. 2014; Chernyakova and Eldar 2014; Zhang et al. 2010; Ng et al. 2006; Belaid et al. 2011). A set of algorithms under signal processing category based on spatial and frequency domains improves visibility.

Spatial domain filtering techniques such as linear, adaptive linear filtering, adaptive Wiener, median, anisotropic diffusion, constraint least mean squares and higher order filtering applied for speckle reduction (Byram et al. 2013; Loizou et al. 2012; Gavriloaia and Gavriloaia 2011; Christos and Constantinos 2008; Yeoh and Zhang 2006). These algorithms did a great job on improving ultrasound images in early days of ultrasound detections. The spatial domain filtering lessens noise inducing a blur to the objects in the ultrasound images. Filter coefficient selection is a difficulty faced by these spatial filtering algorithms.

Pixel based likelihood approaches (Zhang et al. 2007; Yu et al. 2012) such as Bayes classifier Tao Hou et al. (2010) and Gaussian mixture models (GMM) (Gavriloaia and Gavriloaia 2011) denoise algorithms set in the ultrasound scanner. Currently most real time scanners around the world employ these algorithms. Computing the probability density functions and joint probability density functions classify noisy pixels and object pixels in ultrasound images. The probability based algorithms are a little low on accuracy. The denoised ultrasound images in the ultrasound machine still have noise. Their effectiveness loses ground because of the speckle ingredient in ultrasound image varies rapidly between successive images.

Other pixel processing method that revolutionized image processing is thresholding. Thresholding reduces noise from medical ultrasound images by putting a constraint on selection of correct threshold (Achim et al. 2001). However thresholding drops the visual quality of the objects in the image (Trinh et al. 2014). The worst hit parts are edges of objects in the medical image. Edge detection and contrast enhancement are two most popular thresholding methods used on images for visual quality improvement. These processing algorithms suffer dearly when there is a slight difference in sensitivity between pixel intensities of noise and edges (Shaimaa et al. 2012; Lee et al. 2012).

Frequency domain processing of ultrasound images involves filtering of speckle noise in transformed domain (Andria et al. 2013; Wei et al. 2013, 2014). The wavelet transform is exclusively used for speckle reduction. The multiresolution filter bank approach frames computing fast 2D wavelet transform (Dantas and Costa 2007; Rabbani et al. 2008). Filter banks work well at reducing speckle in ultrasound medical images. There are quite a few problems associated with wavelet approaches such as decrease image resolution at higher levels, choice of mother wavelet and loss of edge at higher levels of decomposition (Esakkirajan et al. 2013). Different algorithms are proposed in literature to overcome these effects in recent times showing little enhancements to visual quality (Adamo et al. 2013). Artificial intelligence methods such as artificial neural networks (ANN) (Andria et al. 2012), fuzzy logic (Park and Nishimura 2007), genetic algorithms (Zhang et al. 2010) and ant bee colony algorithm deal with the speckle intelligently (Munteanu et al. 2008). ANN and Fuzzy need extensive training to perform the task on larger data sets. These algorithms give better visual quality only when trained with larger data sets. However, difficulties increase due to the continuously varying nature of speckle in the medical image.

Finally, model based techniques are introduced to produce 3D ultrasound imaging (Fenster and Downey 2013; Latifoglu 2013). This reduced the noise to large extent improving the visibility of objects in the medical image. But these improvements come at a higher price. For most of the poorer countries, it is a matter of affordability. Hence, even though 3D ultrasound model based images is exclusively used in practice it is still difficult to find in a country like India. Hence the speckle reduction in ultrasound medical images will be a major research area in the coming years.

This research paper proposes a novel two fold processing method to reduce the effect of speckle in ultrasound medical images (Huang et al. 2009; Gao and Bui 2005; Rui et al. 2007; Yu et al. 2001). The proposed method calculates the wavelet coefficients from medical image using a multiresolution filter bank approach. The coefficients scaling of amplitude is soft and hard thresholding. Wavelet based object edge reconstruction on the thresholded medical images by using fusion technique is proposed. The wavelet based fusion acts as a value addition to thresholded images to restore the edges of objects in the ultrasound image. This twofold algorithm reduces speckle noise and restores edge quality for better and faster diagnostics by doctors. Verification of the proposed method by doctors at AMMA Hospital, Vijayawada, INDIA and NRI Medical college Hospital, Guntur, INDIA were initiated.

The rest of the paper is organizes as follows. “Twofold proposed technique” gives twofold technique using wavelet transform. “Results and discussion” discusses the results of the proposed algorithm on ultrasound medical image of fetus obtained from AMMA hospital Vijayawada. “Conclusion” compares the results from the proposed algorithm with the results from standard denoising algorithms on medical images. Section 5 concludes the proposed research based on experiments conducted in the previous sections.

## Twofold proposed technique

The two fold technique proposed involves a twostep process in wavelet domain. First step is block thresholding of ultrasound medical image wavelet coefficients followed by fusion of thresholded image with the original image. Thresholding employs hard and soft wavelet thresholding on detailed wavelet coefficients (Marsousi et al. 2013). Apart from removing speckle they also blur the edges. The fusion in wavelet domain restores lost edges of objects during the thresholding. Fusion also improves the contrast of the denoised image. Here adaptive block fusion ensures correct fusion rule at a particular level preserves object properties such as edge and contrast. An ultrasound medical image *U*(*x*, *y*), where $x,y\in Z^\dagger$ and $U\in R^\dagger$ is convolved with a standard orthogonal 2D filter coefficients $f_{s{1}s{2}}^L(x,y)$,where $s_{1}$ and $s_{2}\subset R^\dagger$ denote the scaling factors and $L\subset Z^\dagger$ is decomposition level to produce a 2D discrete wavelet transform having approximate and detailed coefficients as in Eqs. (1) and (2).

The 2D DWT approximate coefficients for a 2D ultrasound signal *U*(*x*, *y*) is formulated as

1 $$\begin{aligned} A_{b}^L=\sum _{i=1}^{b} U_{i}(x,y)\times f_{s_{1}s_{2}}^{IL}(x,y) \end{aligned}$$

And the detailed coefficients are formulated as

2 $$\begin{aligned} D_{b}^L=\sum _{i=1}^{b} U_{i}(x,y)\times f_{s_{1}s_{2}}^{hL}(x,y) \end{aligned}$$

The wavelet decomposition level ‘$L$’ iterates breaking up the image into various frequency surfaces. ‘$l$’ gives low frequency items of the filter and ‘$h$’ are the high frequency items of the filter. Finally ‘$b$’ stands for block size.

The noise in the ultrasound images is found around a few wavelet coefficients. When compared to wavelet object coefficients in the ultrasound image, they are present in large coefficients. Edges mark the boundaries of objects in the image. Thresholding in wavelet domain is making the smaller noise coefficients negligible and larger edge coefficients important. Thresholding of wavelet coefficients reduces speckle noise. However this affects tissue edges that are objects in the denoised images. The edge appears blurred making visually difficult to understand object boundaries.

Therefore global thresholding of wavelet coefficients results in edge loss of objects in the image. Edge loss represents blurring of the edges and decrease in contrast of the ultrasound image as a whole. This can be avoided to a certain extent using the block based thresholding of wavelet coefficients. Block processing makes the thresholding local to that particular block and preserving the contrast in the ultrasound images. Two classes of thresholding algorithms are used to filter wavelet coefficients. They are Hard Thresholding (HT) and Soft Thresholding (ST) as discussed below.

### Block based hard thresholding (BHT)

Block based Hard Thresholding (BHT) is applied on detailed wavelet coefficients of ultrasound image using the expression

3 $$\begin{aligned} D_{bht}^L(i,j)={\left\{ \begin{array}{ll}D_b(i,j)\quad &{}if|D_b(i,j)| > T^L_{bh}\\ 0 \quad &{}if |D_b(i,j)| \le T^L_{bh}\end{array}\right. } \end{aligned}$$

where $D_{bht}^L$ contain the hard threshold wavelet coefficients at locations (i,j) at level. $T_{bh}^L$ is the block threshold value for a particular block of size $b=\lambda _{1}\times \lambda _{2}$ where $\lambda _{1},\lambda _{2}\in Z^\prime ,Z^\prime$ be any positive even number. $T_{bh}^L$ is computed for each block using the expression.

4 $$\begin{aligned} T_{bh}^L=\sum _{i=1}^{\lambda 1}\sum _{j=1}^{\lambda 2} D_{b}^L(i,j)/M \end{aligned}$$

M is the maximum number of gray levels in the original image $U_{i}(x,y)$.

### Block based soft thresholding (BST)

Block based soft thresholding is defined according to, the soft threshold value $T_{bs}^L$ in each block is computed as

5 $$\begin{aligned} T_{bs}^L=\xi \sqrt{2log(m)} \end{aligned}$$

where M is the number of pixels in the image and $\xi$ gives

6 $$\begin{aligned} \xi _{b}^A=\frac{|median(U_{b}(x,y)) |}{0.6745} \end{aligned}$$

$\xi$ on detailed wavelet coefficients estimates to

7 $$\begin{aligned} \xi _{b}^D=\frac{|median(D_{b}^L)| }{0.6745} \end{aligned}$$

Block based Soft thresholding (BST) on detailed wavelet coefficients using the equation

8 $$\begin{aligned} D_{bst}^L(i,j)={\left\{ \begin{array}{ll}sgn(D_b^L(i,j))\times (|D_b^L(i,j)|-T_{bs}^L) \quad &{}if |D_b^L(i,j)|>T_{bs}^{LD}\\ 0 \quad &{}if |D_b^L(i,j)|\le T_{bs}^{LD}\end{array}\right. } \end{aligned}$$

where $D_{bst}^L(i,j)$ are soft thresholded coefficients at level L at location (i,j).Where sgn(n) is a signum function. The inverse transformation using the low pass and high pass reconstruction filters results in a quality image $U^{(d)}(x,y)$. However, closer observation of denoised images shows blocking artifacts at some locations on the image. This happens when the block size $b=\lambda 1 \times \lambda 2$, where $\lambda 1,\lambda 2 \in Z^\prime$ , is small comparable to the size of the original ultrasound image. Though thresholding in wavelet domain removes speckle well with blurring of the region of interest objects.

The proposed solution for removing blocking artifacts and blurring of region of interest objects comes from wavelet based fusion. Fusion in wavelet domain improves the visual quality of the degraded images from multiple sources. The second technique is fusion of the original ultrasound medical image and the denoised ultrasound image from the first technique in wavelet domain.

The fusion aims to combine wavelet coefficients of block denoised US image $U^{(d)}(x,y)$ with original ultrasound medical image U(x,y).The coefficients of different blocks fuse together by selection of fusion rules and levels in wavelet for each block. Wavelet level select and fusion type are selected based on the properties of object strength present in the blocks. The object strength parameter is edge strength (Gao and Bui 2005) of each denoised block.

Edge strength is most widely used in image processing to measure the quality edge detection algorithms (Gao and Bui 2005). Here it measures the strength of edges in the original US image which contribute towards object characteristics. Two D gradient operator calculates the edge magnitude $\epsilon (x,y)$and edge orientation $\theta (x,y)$ for each pixel in the block. For the original ultrasound image U(x,y),it is defined as

9 $$\begin{aligned} \epsilon ^b(x,y)=\sqrt{g_{x}^b(x,y)^2+g_{y}^b(x,y)^2} \end{aligned}$$

10 $$\begin{aligned} and \,\,\,\, \theta ^b(x,y)=\tan ^{-1}(\frac{g_{y}^b(x,y)}{g_{x}^b(x,y)}) \end{aligned}$$

where $\epsilon ^b(x,y)$ and $\theta ^b(x,y)$ provide edge information and edge orientation respectively of each block b. $g_{x}^b(x,y)$ and $g_{y}^b(x,y)$ are block gradients along x and y directions. The next step computes histogram of magnitude $h_{gm}^b(x,y)$ and orientation $h_{g\theta }^b(x,y)$ for the original ultrasound image. The histograms of gradient (Bhuiyan et al. 2009) blocks give the magnitude and orientation of pixels marking edges of objects in the block.

Comparing the histograms of adjacent blocks magnitude and orientation will disclose the presence of object. For comparison of gradient histograms a parameter called normalized differential mean (NDM) is computed on the adjacent blocks. The expression for NDM for two gradient magnitude histograms is

11 $$\begin{aligned} N_{\theta }^{b(K,K+n)}=\sum _{i\epsilon b}\frac{|h_{g\theta }^{i(k)}(x,y)-h_{g\theta }^{i(k+n)}(x,y)| }{||h_{g\theta }^{i(k)}(x,y)+h_{g\theta }^{i(k+n)}(x,y)|| } \end{aligned}$$

12 $$\begin{aligned} N_{\theta }^{b(K,K+n)}=\sum _{i\epsilon b}\frac{|h_{g\theta }^{i(k)}(x,y)-h_{g\theta }^{i(k+n)}(x,y)| }{|| h_{g\theta }^{i(k)}(x,y)+h_{g\theta }^{i(k+n)}(x,y)|| } \end{aligned}$$

$$N_{\epsilon }^{b(K,K+n)}$$ and $$N_{\theta }^{b(K,K+n)}$$ denote the normalized differential means of gradient magnitude histogram and gradient orientation histogram between $$K^{th}\, {\rm and} \,(K+n)^{th}$$ blocks for each pixel $$i\in (b\subseteq \lambda 1, \lambda 2)$$ within the block. The values $$N_{\epsilon }^{b(K,K+n)}$$,$$N_{\theta }^{b(K,K+n)}$$$$\subset$$$$R^2$$ belong to a set of positive real numbers between 0, 1. The extreme valve of 0 shown no difference between the two adjacent blocks whereas orthogonality between blocks is indicated the value of 1. The degree of object presence in a particular block is indicated by a value close to 1.

**Fig. 1:**
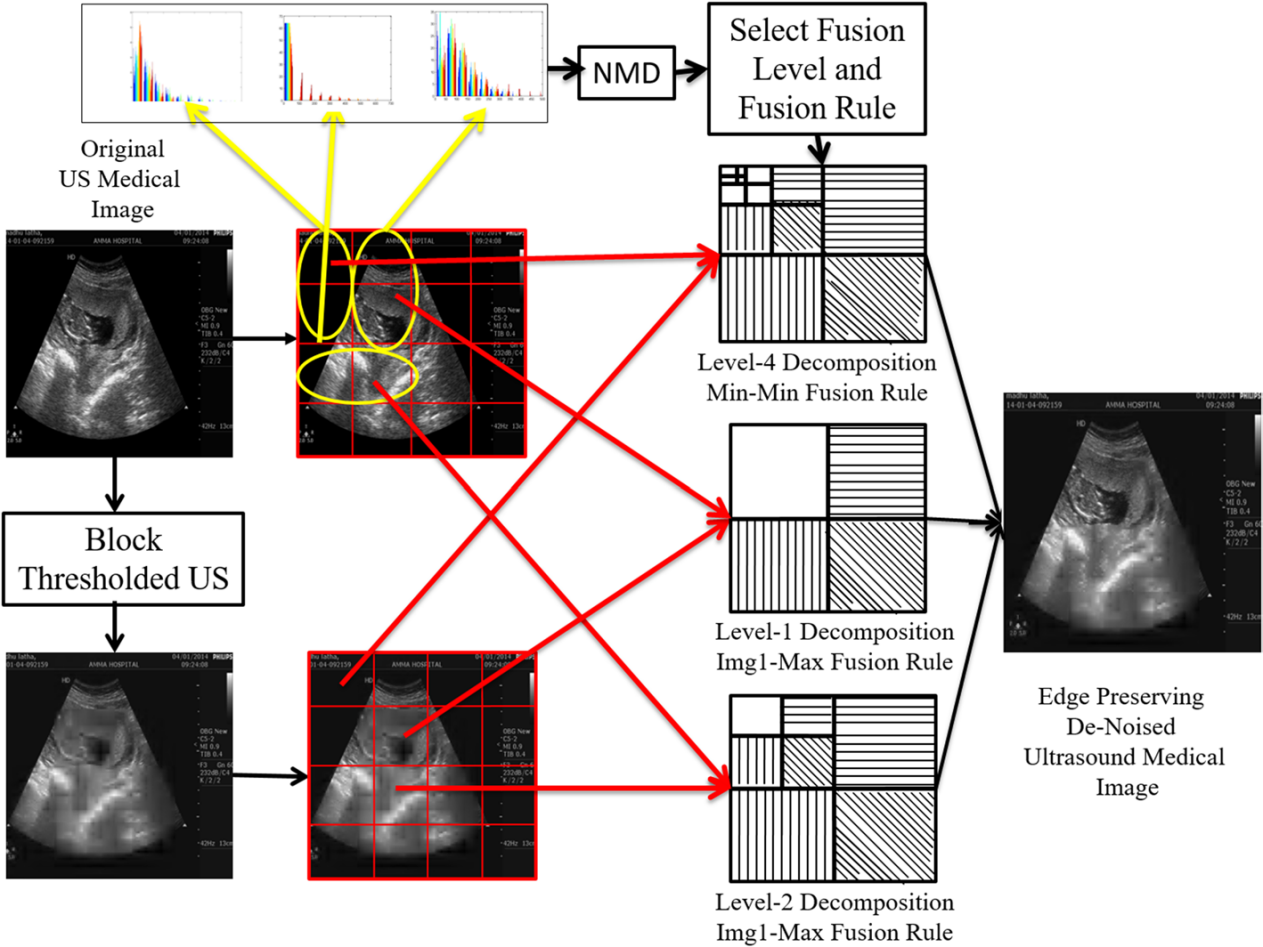
Proposed fusion process for level selection and rule selection for ultrasound medical image de-noising in wavelet domain

Each block of original ultrasound image U(x,y) and denoised ultrasound image $U^(d)(x,y)$ are fused at various levels and with different fusion rules based on the magnitude and orientation values. The complete picture of the entire de-noising process is represented in the Fig. 1.

Adjacent blocks are checked to select the fusion level and fusion rule from a set of five fusion levels and eight fusion rules in wavelet domain.

The five fusion levels are named as L1, L2, L3, L4 and L5. Fusion rules select approximate and detailed wavelet coefficients for fusion from their respective levels. Eight fusion rules are represented as $F^{(A_{max},D_{max})},F^{(A_{min},D_{max})},F^{(A_{max},D_{min})},F^{(A_{min},D_{min})}, F^{(A_{img1},D_{max})},F^{(A_{img1},D_{min})},F^{(A_{img2},D_{max})},F^{(A_{img2},D_{min})}, F^{(A_{max},D_{max})}$fusion rule selects approximate maximum coefficients and detailed maximum coefficients from original ultrasound medical image U(x,y) and block de-noised ultrasound medical image $U^{(d)}(x,y)$ with hard and soft thresholding in wavelet domain. Remaining fusion rules are defined in literature (Di Huang et al. 2014) and selected accordingly. The following fusion mechanisms are employed for various values of $N_{\epsilon }^b$ and $N_{\theta }^b$ in the range of 0–1 between blocks

13 $$\begin{aligned}{}[F^{(A_{img1,D_{max}})},L_{1}]\Leftarrow 1\le (N_{\epsilon }^b,N_{\theta }^b)\le 0.955 \end{aligned}$$

14 $$\begin{aligned}{}[F^{(A_{img1,D_{min}})},L_{1}]\Leftarrow 0.954\le (N_{\epsilon }^b,N_{\theta }^b)\le 0.855 \end{aligned}$$

15 $$\begin{aligned}{}[F^{(A_{img2,D_{max}})},L_{2}]\Leftarrow 0.854\le (N_{\epsilon }^b,N_{\theta }^b)\le 0.755 \end{aligned}$$

16 $$\begin{aligned}{}[F^{(A_{img2,D_{min}})},L_{2}]\Leftarrow 0.754\le (N_{\epsilon }^b,N_{\theta }^b)\le 0.655 \end{aligned}$$

17 $$\begin{aligned}{}[F^{(A_{min,D_{min}})},L_{3}]\Leftarrow 0.654\le (N_{\epsilon }^b,N_{\theta }^b)\le 0.555 \end{aligned}$$

18 $$\begin{aligned}{}[F^{(A_{min,D_{min}})},L_{4}]\Leftarrow 0.554\le (N_{\epsilon }^b,N_{\theta }^b)\le 0.455 \end{aligned}$$

19 $$\begin{aligned}{}[F^{(A_{min,D_{min}})},L_{5}]\Leftarrow 0.454\le (N_{\epsilon }^b,N_{\theta }^b)\le 0.001 \end{aligned}$$

Inequalities 13 to 19 are the proposed new set of fusion rules based on selection of levels and fusion rules. Figure 1 shows the astonishing improvement in ultrasound image quality by reducing the noise component in the image. From the Fig. 1 it can be observed that the fused de-noised image $U_{f}^{(d)}(x,y)$ is visually far superior quality compared to the ultrasound images on the left of the Fig. 1, which are original ultrasound and thresholded ultrasound images.

Testing of the proposed de-noising method to remove multiplicative speckle from the onsite ultrasound medical images procured from AMMA hospital radiology department. The fetus images are obtained in consultation with their doctors by agreeing upon all legal matters as per the constitution of government of India

## Results and discussion

Testing of the proposed method for speckle reduction on ultrasound medical images has to be accomplished by measuring the visual excellence. The parameters that are trusted with this job are peak signal to noise ratio (PSNR), normalized cross correlation coefficient (NCC), edge strength, image quality index (IQI), and structural similarity index (SSIM) (Yu et al. 2001; Lanzolla et al. 2011; Wang and Bovik 2002; Wang et al. 2004).The following popular denoising algorithms from literature that are most likely used for speckle reduction are anisotropic diffusion (AD) (Farias and Akamine 2012), Total Variational Filter (TVF) (Yoon et al. 2012) and Empirical mode decomposition (EMD) (Hu and Jacob 2012). Our proposed algorithm is tested against these techniques both visually and measurably.

For experimentation on ultrasound medical images are procured from two hospitals in Vijayawada, Andhra Pradesh, India. They are AMMA hospitals and NRI medical college hospital. The doctors are consulted and legal agreements are signed as per Indian constitution for sharing ultrasound fetal medical information. And more over doctors helped in detecting and gauging the visual quality of the proposed method with remaining filtering methods in extracting the information from the ultrasound scans. Time for information extraction from filtered images is noted to find the importance of applying this method for clinical application.

**Fig. 2:**
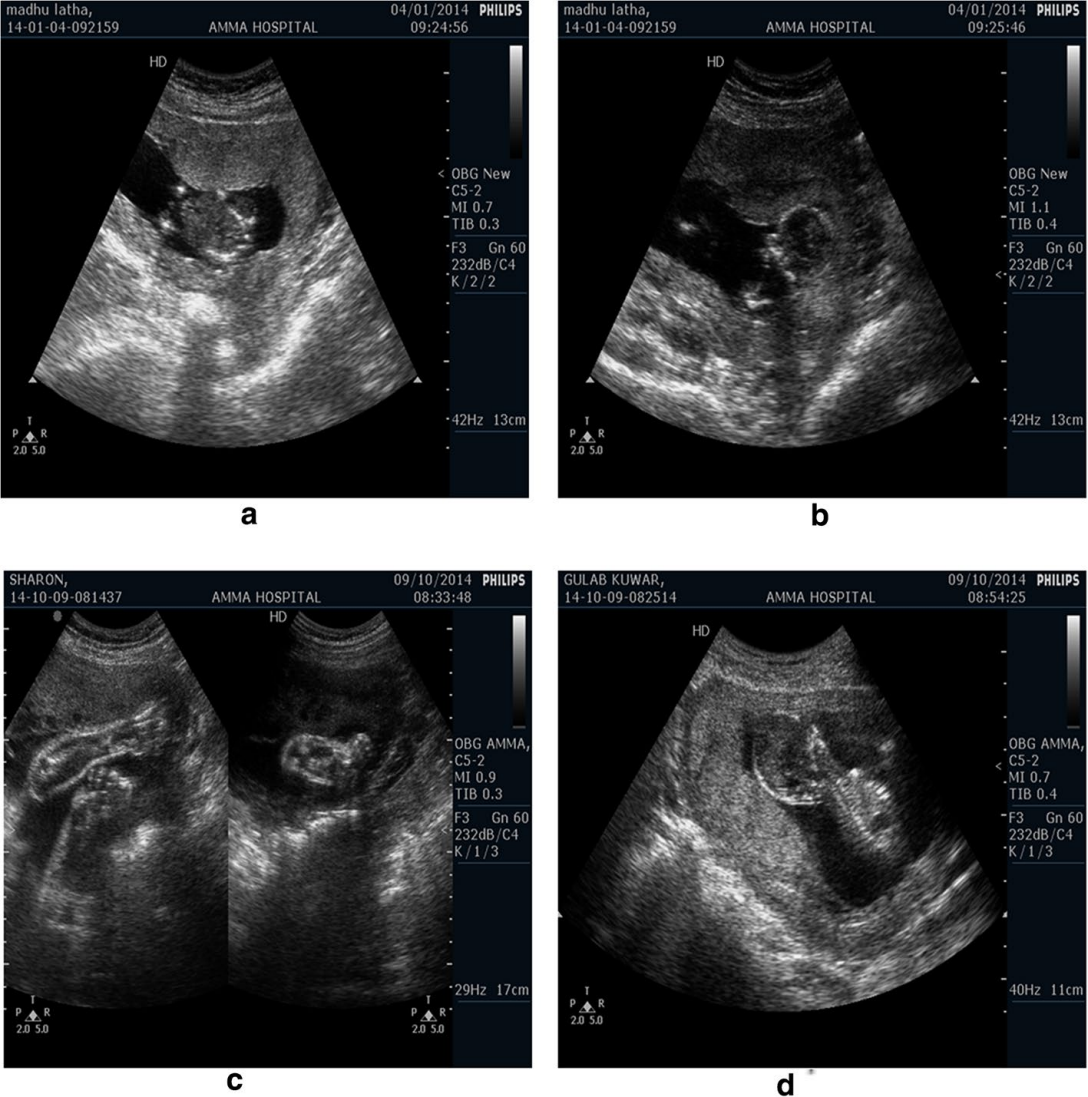
**a**–**d** Fetus Ultrasound Images captured at radiology lab of AMMA hospital of various patients

The images used for experimental testing of proposed speckle reduction technique are fetus ultrasound images. These images contain fetus of women at various stages of pregnancy. A total of 4 ultrasound images are used for experimentation. These images are generated by 4 ultrasound machines from Philips excited with 42Hz sonographic sound and response imaging display of 13 cm as shown in Fig. 2. Images are converted from machine specific imaging format to tagged image file format (tiff) with 8 bit sampling rates. Images are normalized to standard resolution of $256\times 256$.

**Fig. 3:**
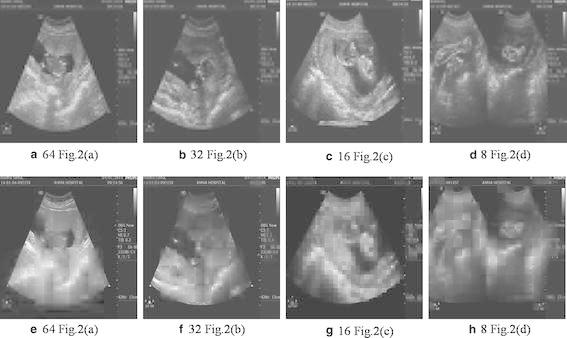
Processed US images of original images from 2(a)–2(d) using (**a**–**d**) Block Hard Thresholding with block sizes 64,32,16 and 8, **e**–**h** Block Soft Thresholding with block sizes 64,32,16 and 8

The following block sizes of 8, 16, 32 and 64 divides the pixels into standard blocks. DWT translates each block from spatial domain to wavelet domain. Hard Thresholding (BHT) on detailed wavelet coefficients using the Eq. (4) gives adjusted coefficients.The approximate coefficients show smooth variation and are hence un-thresholded. The individual blocks having approximate and thresholded detailed components are inverse transformed to spatial domain. Finally, concatenation of all the blocks gives a denoised ultrasound spatial domain medical image. Only 8, 16, 32 and 64 blocks provided good denoising for a standard $256\times 256$ resolution image. Less than 8 and greater than 64 block size, consume processer time and with very little influence on the end result respectively. Figure 3a, b show the result of denoised ultrasound fetus images of Fig. 2a, b for a block size of 16.

Similarly, block soft thresholding (BST) reduces the speckle using eq’n (8) applied of detailed coefficients of each block. Figure 3c, d provides results of soft thresholding. Observing the resultant images in Fig. 3, show a smooth variation among pixels in case of soft thresholding compared to hard thresholding. Compared with original US images, visual improvement is noticeable in the resulting images. But, the trained doctors at AMMA hospital did not show much of an interest in the images of Fig. 3. The reason is blurring of objects of interest resulted due to higher thresholds of wavelet coefficients and not much of a difference observed for lower thresholds. A suggestion from the practicing doctors of US imaging is to improve contrast between the object of interest and the remaining portions of the image so that they can have better visual information and can help faster detection. Their point was to high light the object of interest region with less noise removal and smoothing the remaining portions resulting in a high contrast US image. Hence the second fold processing on the block thresholded denoised images is initiated.

**Fig. 4:**
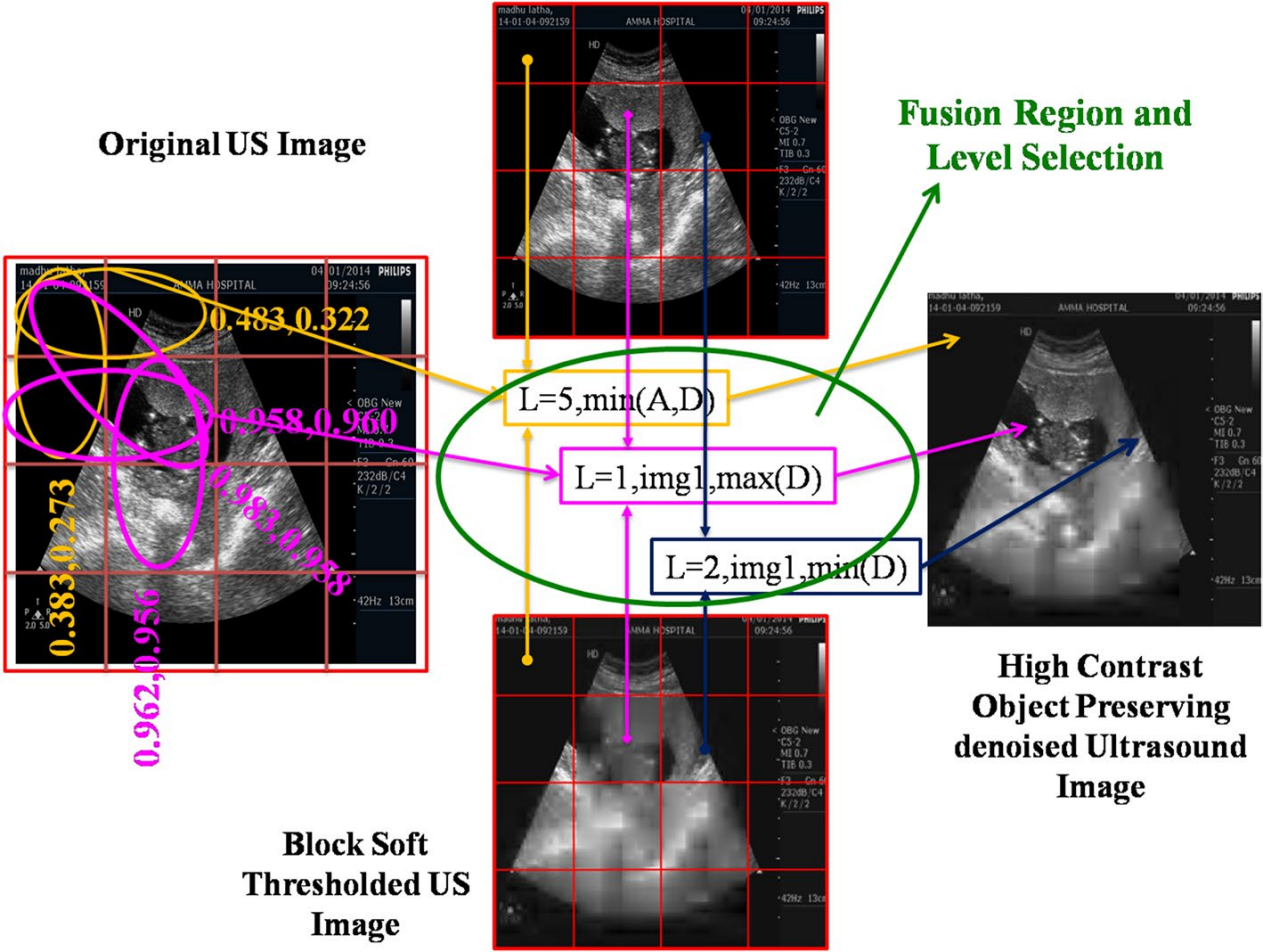
Fusion algorithm for developing denoised high contrast ultrasound images

In the second fold each block of denoised image in the first fold compares with 8 adjacent blocks to identify a valid edge and its orientation. Normalized differential means of histogram gradient values selects the wavelet level and fusion rule. For the original ultrasound image in Fig. 2a we apply a 64 block denoising using soft thresholding technique to obtain Fig. 3e in the first fold. The second fold begins with identifying edge containing blocks from original US image in 2(a). In this the $256\times 256$ is divided into 16 blocks of each $64\times 64$. Computing histogram of gradients on each $64\times 64$ block and extracting mean magnitude and mean orientations on adjacent blocks. The mean values are shown in the Fig. 4 for as an example. Magnitude and angle mean of 1st $64\times 64$ block and it’s neighbors produces the values imprinted on left figure in Fig. 4. The first two horizontal blocks compared in mean histogram gradient $(N_{\epsilon }^b,N_{\theta }^b)$ took values (0.483,0.322).similarly for vertical and diagonal neighborhoods the values are (0.383,0.273) and (0.983,0.273) respectively. These values help to detect the presence of edges in a block and to restore these edges in that particular block from the original ultrasound image through fusion. Fusion is performed in wavelet domain. The type of fusion and wavelet decomposition level for fusion depend on the mean gradient histogram values.The rules for fusion are as in eq’s 13-19. Eight fusion rules and 5 levels of decomposition are used.

As in Fig. 4 the 1st $64\times 64$ block does not have edges and hence select level 5 wavelet decomposition (L = 5) with minimum value coefficients from approximate and detailed components for fusion. From Fig. 4 it can be seen that the object of interest occupies block (2, 2). The mean histogram values with neighboring blocks for this block are very high (0.983, 0.958). The information seems important to the user and hence L = 1 and approximate components from original Ultrasound image and maximum of details by comparing both the original US and Denoised US images. It is clearly observable that compared to denoised image the fusion based denoised image gives object of interest clarity.

**Fig. 5:**
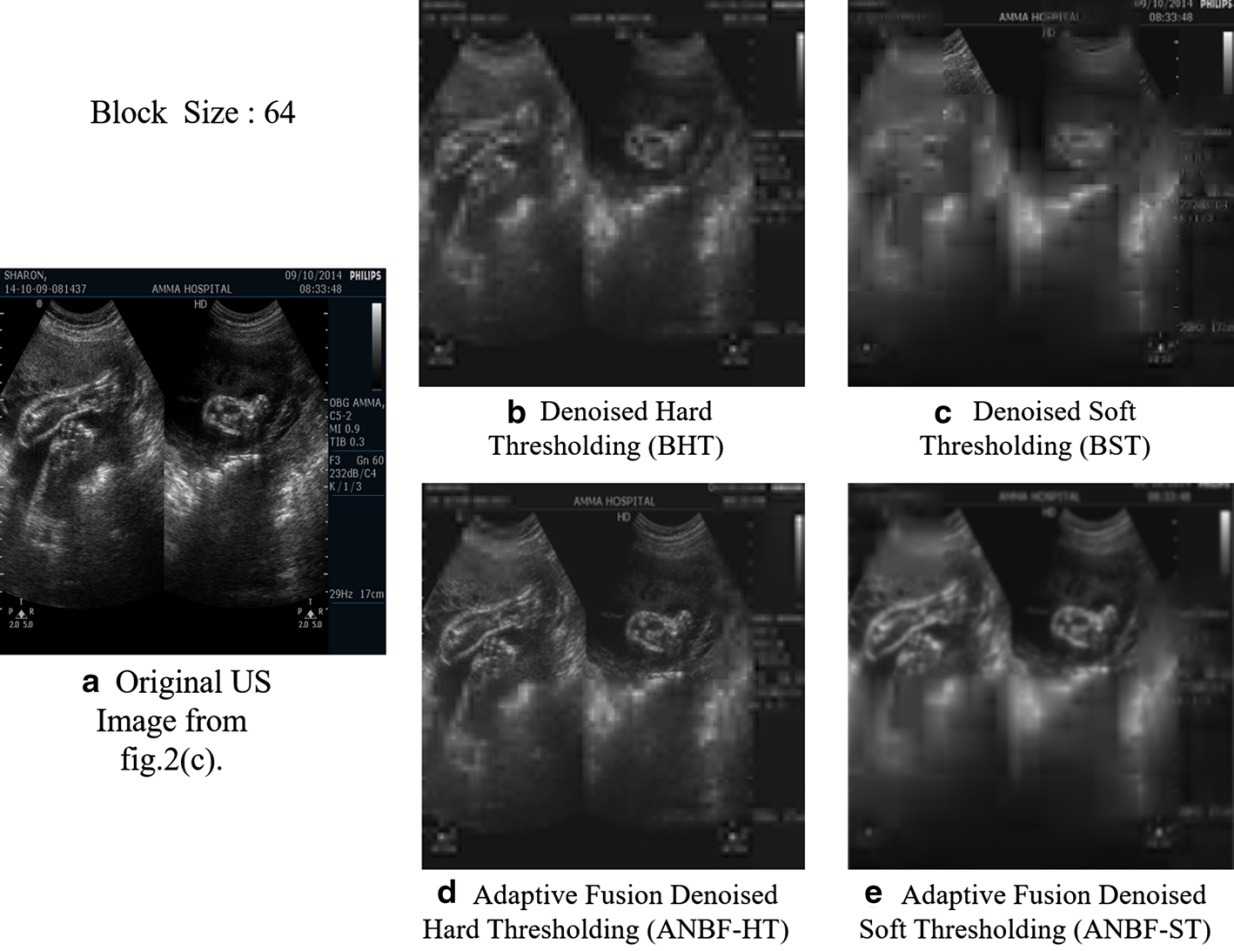
Comparison images of visual quality for block hard and soft thresholding and two fold processing methods for a block size of 64 **a** Original US image from 2(c), **b** BHT, **c** BST, **d** Adaptive Fusion Hard Thresholding (ANBF-HT), **e** Adaptive Fusion soft thresholding (ANBF-ST)

Here we show the visual clarity of the proposed two fold denoising against block hard and soft thresholding for the image Fig. 2c. Object clarity in the denoised image with high contrast helps detect and analyze the two fold denoised images in short time. Figure 5 showing original US image in 5(a) from Fig. 2c, denoised image BHT in Fig. 5b, BST in Fig. 5c, Adaptive fusion with HT (ANBF-HT) in Fig. 5d and Adaptive fusion with ST (ANBF-ST) in Fig. 5e.

**Fig. 6:**
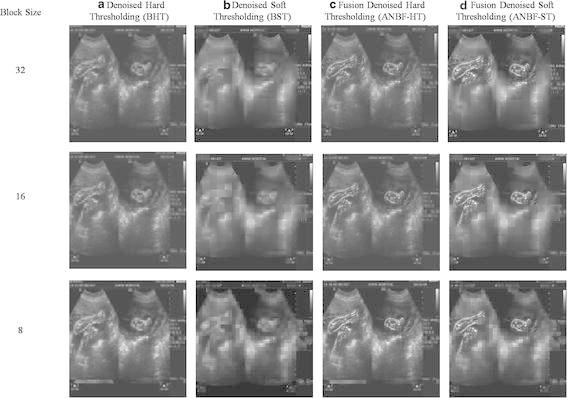
32,16 and 8 block Comparison images having columns **a** BHT , **b** BST, **c** Adaptive Fusion Hard Thresholding (ANBF-HT), **d** Adaptive Fusion soft thresholding (ANBF-ST)

Visually the two fold denoised images preserve objects and show good contrast between the object boundaries and the rest of the image. Figure 6 shows the denoising methods for block sizes 32, 16 and 8.

Increasing non overlapping block size results in increasing contrast but introduces blocking artifacts that tend to distract observations at high resolutions above 512 pixels. But at medium resolutions such as $256\times 256$, blocking artifacts does not influence quality. Table 1 show the fusion rules and levels for the image in Fig. 5. There will 16 blocks for a $256\times 256$ US image. Table 1 gives the happenings on each block during denoising.

**Table 1:** Level and Fusion rule selection based in Eqs. (11–19) for the image in Fig. 4 using ANBF-HT

Block no	$(N_{\epsilon }^b,N_{\theta }^b)$	Level	Fusion rule
1	0.181,0.102	5	$F(A_{min},D_{min})$
2	0.399,0.322	5	$F(A_{min},D_{min})$
3	0.229,0.213	5	$F(A_{min},D_{min})$
4	0.182,0.101	5	$F(A_{min},D_{min})$
5	0.976,0.979	1	$F(A_{min},D_{min})$
6	0.958,0.950	1	$F(A_{min},D_{min})$
7	0.949,0.922	2	$F(A_{img1},D_{min})$
8	0.637,0.620	3	$F(A_{min},D_{min})$
9	0.425,0.433	5	$F(A_{min},D_{min})$
10	0.543,0.521	4	$F(A_{min},D_{min})$
11	0.523,0.532	4	$F(A_{min},D_{min})$
12	0.282,0.221	5	$F(A_{min},D_{min})$
13	0.388,0.342	5	$F(A_{min},D_{min})$
14	0.422,0.431	5	$F(A_{min},D_{min})$
15	0.412,0.412	5	$F(A_{min},D_{min})$
16	0.199,0.195	5	$F(A_{min},D_{min})$

Denoising quality of the US images can be best assessed using a set of calculations known as denoising quality metrics. These are peak signal to noise ratio (PSNR), image quality index (IQI), normalized cross correlation coefficient (NCC), edge strength (ES) and structural similarity index (SSIM) as in (Rui et al. 2007; Yu et al. 2001; Marsousi et al. 2013). Metrics calculations on considered block sizes for all images of Fig. 2, gives values in Table 2. Divisions in Table 2 show for 4 US images in Fig. 2 with 4 block sizes.

**Table 2:** Quality metrics for test images in Fig. 2 for two fold techniques for various block

US TEST IMAGES Fig. 2	PSNR	NCC	ES	IQI	SSIM
SOFT 81(S81)	25.7301	0.9604	0.5286	0.8303	0.7421
SOFT 82(S82)	33.6710	0.9335	0.5342	0.8287	0.7550
SOFT 83(S83)	28.0827	0.9186	0.8909	0.7659	0.6957
SOFT 84(S84)	23.2421	0.9526	0.5447	0.8615	0.7734
SOFT 161(S161)	31.5440	0.9638	0.6297	0.7742	0.7449
SOFT 162(S162)	32.5186	0.9406	0.6301	0.7822	0.7666
SOFT 163(S163)	40.1493	0.9302	0.9992	0.8414	0.8938
SOFT 164(S164)	22.7073	0.9535	0.6405	0.8022	0.7587
SOFT 321(S321)	25.4860	0.9734	0.8221	0.7058	0.7948
SOFT 322(S322)	31.3533	0.9578	0.8110	0.7113	0.8135
SOFT 323(S323)	33.1039	0.9476	0.9825	0.7177	0.7343
SOFT 324(S324)	25.2114	0.9605	0.8035	0.7157	0.7791
SOFT 641(S641)	30.3625	0.9788	0.9604	0.6544	0.7988
SOFT 642(S642)	39.5023	0.9814	0.8568	0.7480	0.9029
SOFT 643(S643)	30.1731	0.9489	0.9717	0.7089	0.7249
SOFT 644(S644)	38.5144	0.9742	0.8212	0.7365	0.8299
HARD 81(S81)	39.2534	0.9702	0.5146	0.8841	0.7983
HARD 82(S82)	30.4953	0.9452	0.5210	0.8811	0.8054
HARD 83(S83)	27.0829	0.9394	0.8403	0.8655	0.7741
HARD 84(S84)	29.9035	0.9604	0.5338	0.9164	0.8263
HARD 161(S161)	40.2058	0.9726	0.6144	0.8048	0.7962
HARD 162(S162)	30.6303	0.9520	0.6286	0.7955	0.7996
HARD 163(S163)	28.7951	0.9431	0.9561	0.8211	0.7722
HARD 164(S164)	34.4221	0.9665	0.6422	0.8422	0.8188
HARD 321(S321)	32.8812	0.9713	0.8286	0.6834	0.7855
HARD 322(S323)	28.8381	0.9510	0.8284	0.6810	0.7973
HARD 323(S324)	37.5740	0.9434	0.9934	0.7719	0.7752
HARD 324(S324)	33.4592	0.9658	0.8034	0.7441	0.8179
HARD 641(S641)	43.0055	0.9711	0.9187	0.6183	0.7809
HARD 642(S642)	28.5056	0.9566	0.9219	0.8497	0.9388
HARD 643(S643)	29.7116	0.9514	0.9383	0.8129	0.8125
HARD 644(S644)	29.7116	0.9616	0.9419	0.6863	0.8091

From the Table 2, a set of observations will decide on the performance of two fold techniques used for enhancing US images. The observations of our interest are related of speckle reduction given by psnr, edge preserving by ES and SSIM, relativity with originality by NCC and contrast by IQI. Overall performance from the Table regarding proposed method is within the acceptable measures according to ultrasound image denoising research.

**Fig. 7:**
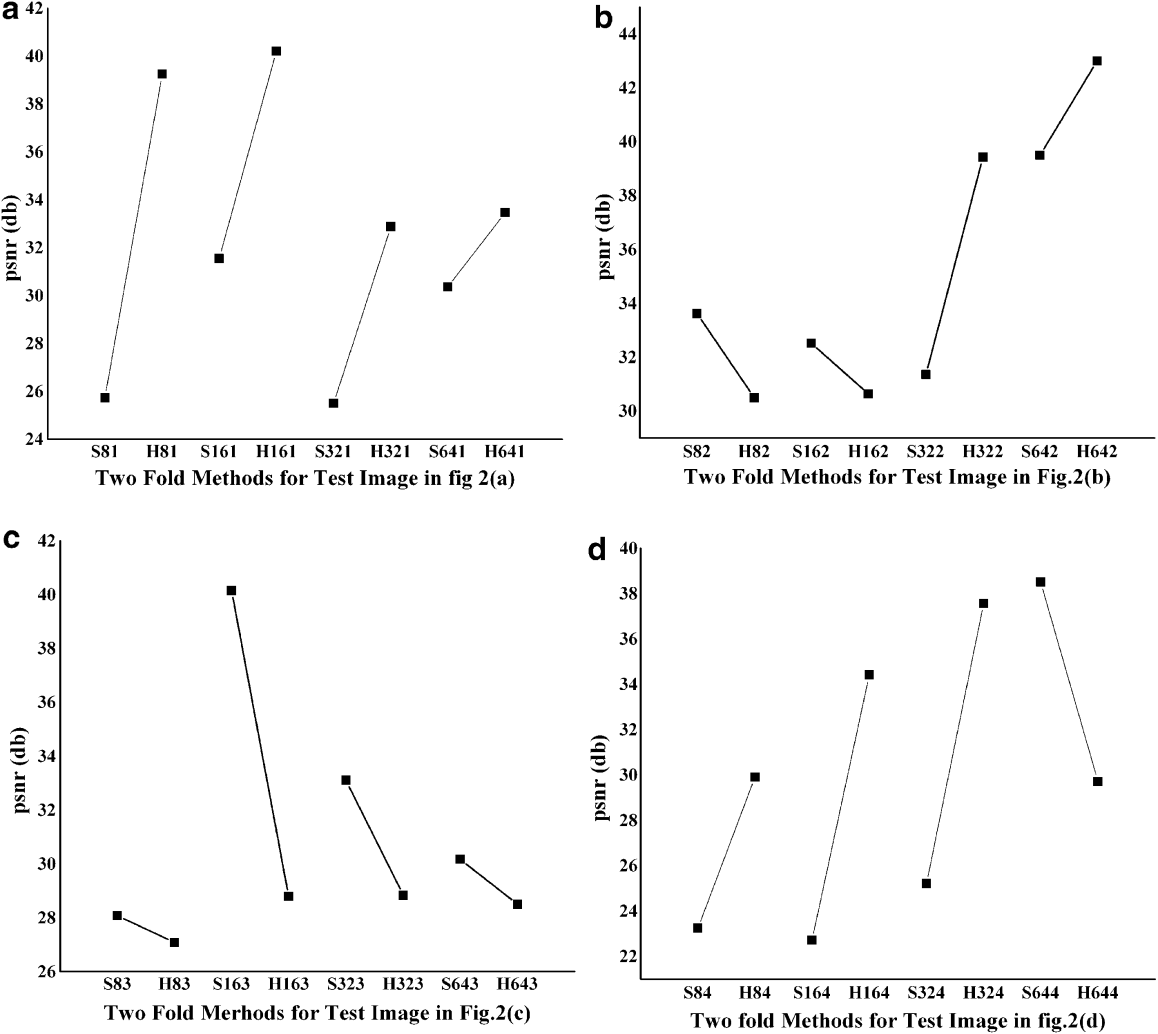
PSNR in db for the test images from Fig. 2 using two fold methods i.e. ANBF-HT and ANBF-ST for block sizes 8, 16, 32 and 64

Figure 7 shows the PSNR in db for the test images in Fig. 2 for two fold processing with hard thresholding (ANBF-HT) and soft thresholding (ANBF-ST).

From the plots in Fig. 7, PSNR for the proposed two fold techniques give mixed results. The first two test images from Fig. 2a, b are from the same patient with a one minute delay in image capture. In the graph of Fig. 7a there is a 100 % domination of ANBF-HT and it gives good PSNR of around 40db at block sizes 8 and 16. For higher block sizes PSNR falls, but under acceptable levels. Coming to Fig. 2b and its PSNR plot in Fig. 7b, there is 50 % domination by the two methods ANBF-HT and ANBF-ST. But ANBF-HT is a clear winner at higher block sizes.

**Fig. 8:**
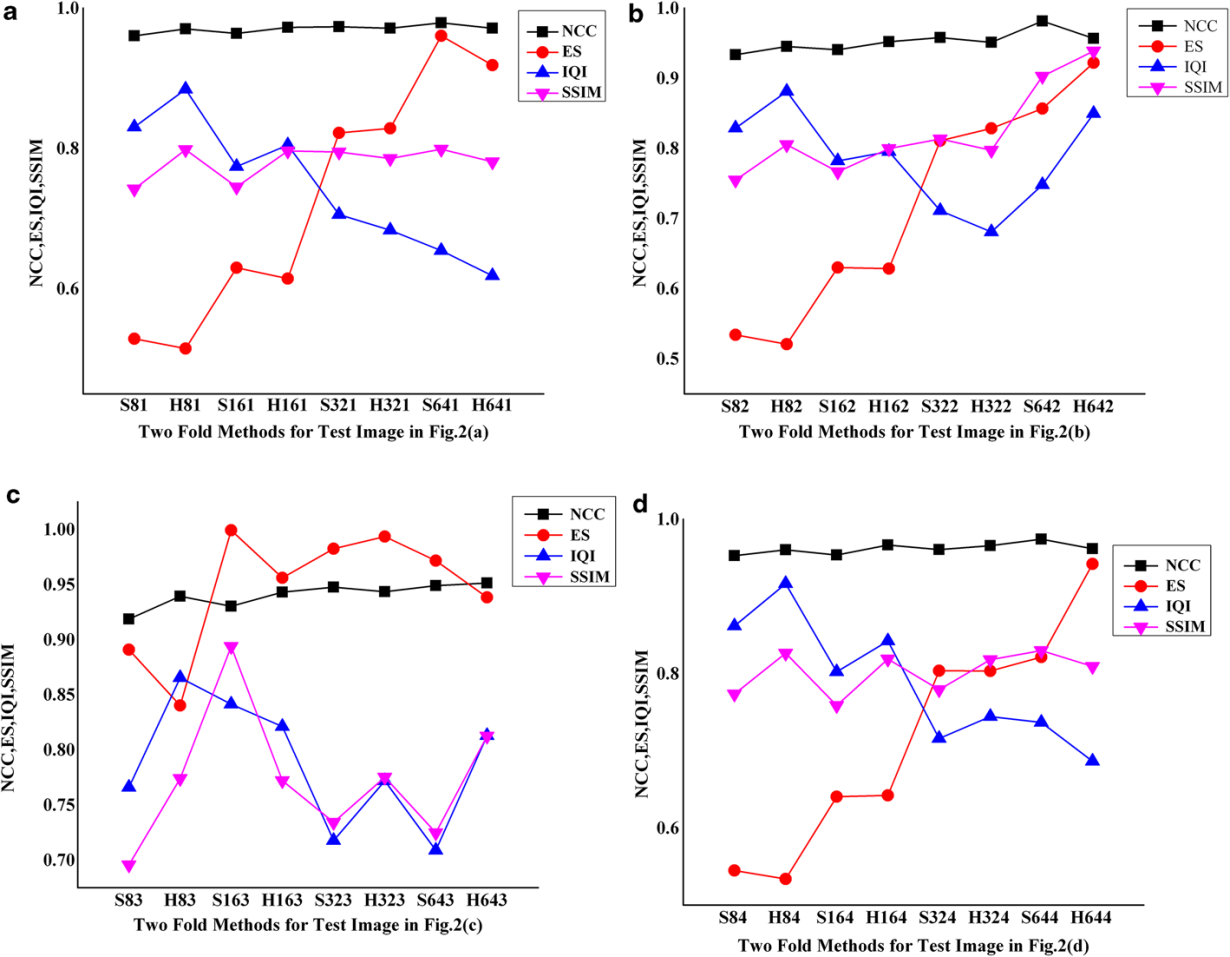
Plots of NCC, ES, IQI and SSIM for Test images in Fig. 2

The above results point towards the characteristics of speckle in real time ultrasound imaging. The reason for variations in block sizes for hard and soft thresholding is in the object structure in the US image. Figure 2a, b, d have good solid edge boundary compared to Fig. 2c. As the two fold technique adaptively selects edge blocks for fusion, hard threshold dominates for preserving sharp discontinuities. Figure 2c is having smooth variation of pixels and hence the PSNR is dominant for soft thresholding (ANBF-ST) as in Fig. 7c. Figure 8a–d provides plots of NCC, ES, IQI and SSIM for the proposed two fold denoising methods.

NCC (Normalized Cross Correlation) is the figure telling the relativity of the denoised image with original US image. Figures close to 1 indicate high correlation. Fig. 8a–d shows a constant NCC value over the entire range of methods and block sizes. This shows that the objects in image are intact after denoising. Edge Strength (ES) is increasing with increase in block size. The reason this characteristic of ES is the presence of thick edges in the original image occupying more than 8 or 16 pixels. IQI (Image Quality Index) falls with block size increase in all the cases and at times fluctuating rapidly between blocks and thresholds as in Fig. 8c. Except for Fig. 8c, SSIM is fairly constant. Figure 2c has smooth edges which are difficult to structure out from the object and hence good SSIM.

All in all the parameters show the proposed methods for denoising retains most of the object characteristics removing speckle, thereby improving visual contrast preserving object boundaries.

**Fig. 9:**
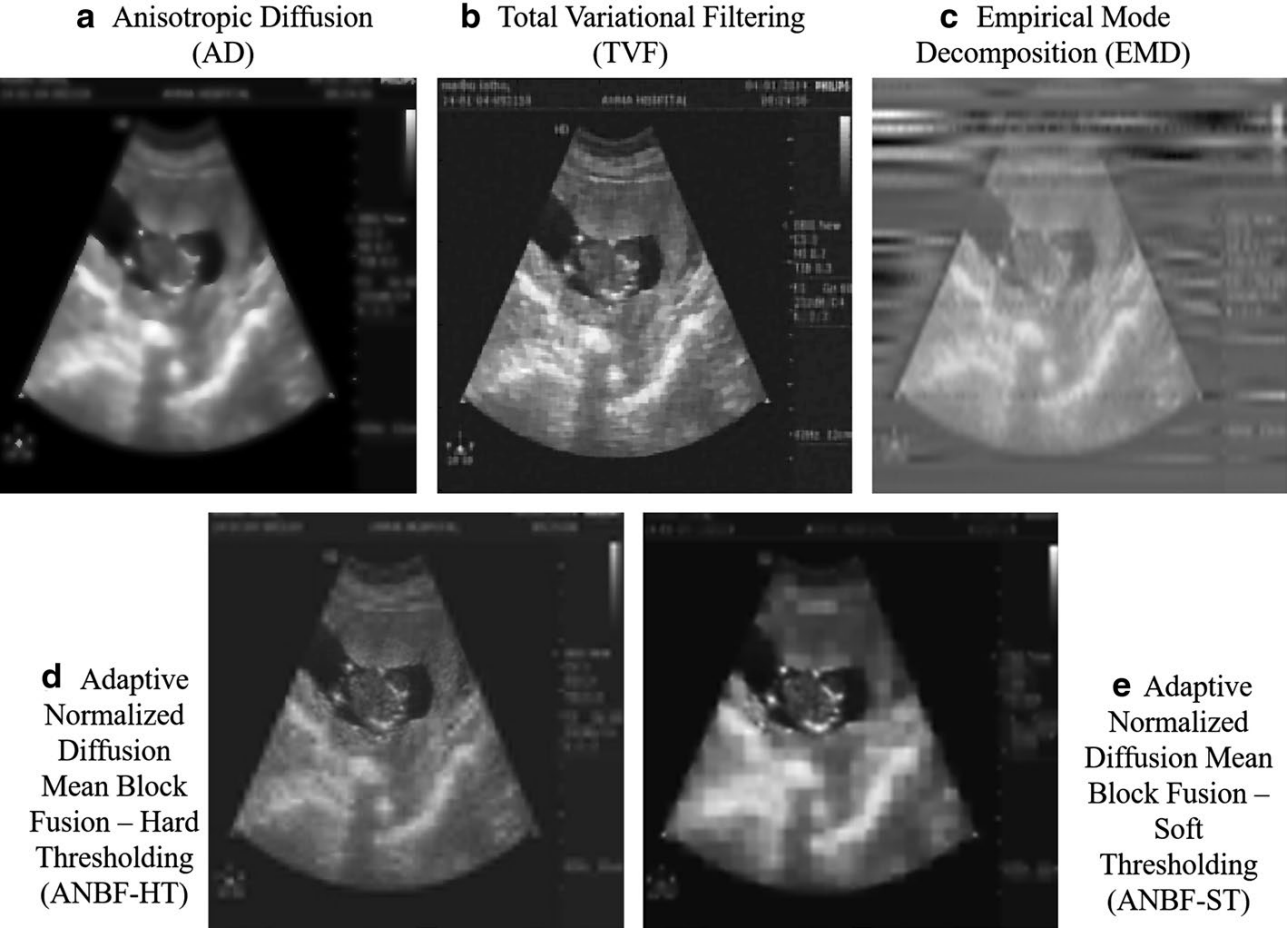
Test image from Fig. 2a denoised using **a** Anisotropic Diffusion with 40 iterations, **b** Total variational Filtering (TVF) with 50 iterations, **c** Empirical Mode Decomposition (EMD) with 5 Modes, **d** Adaptive Normalized Diffusion Mean Block Fusion-HT (ANBF-HT) with block size 16, **e** Adaptive Normalized Diffusion Mean Block Fusion-ST (ANBF-ST) with block size 8

The most famous denoising algorithms of recent times for ultrasound image denoising are Anisotropic Diffusion (AD) and Total Variational Filtering (TVF). Also included a recently proved technique for denoising is Empirical Mode Decomposition (EMD).

**Fig. 10:**
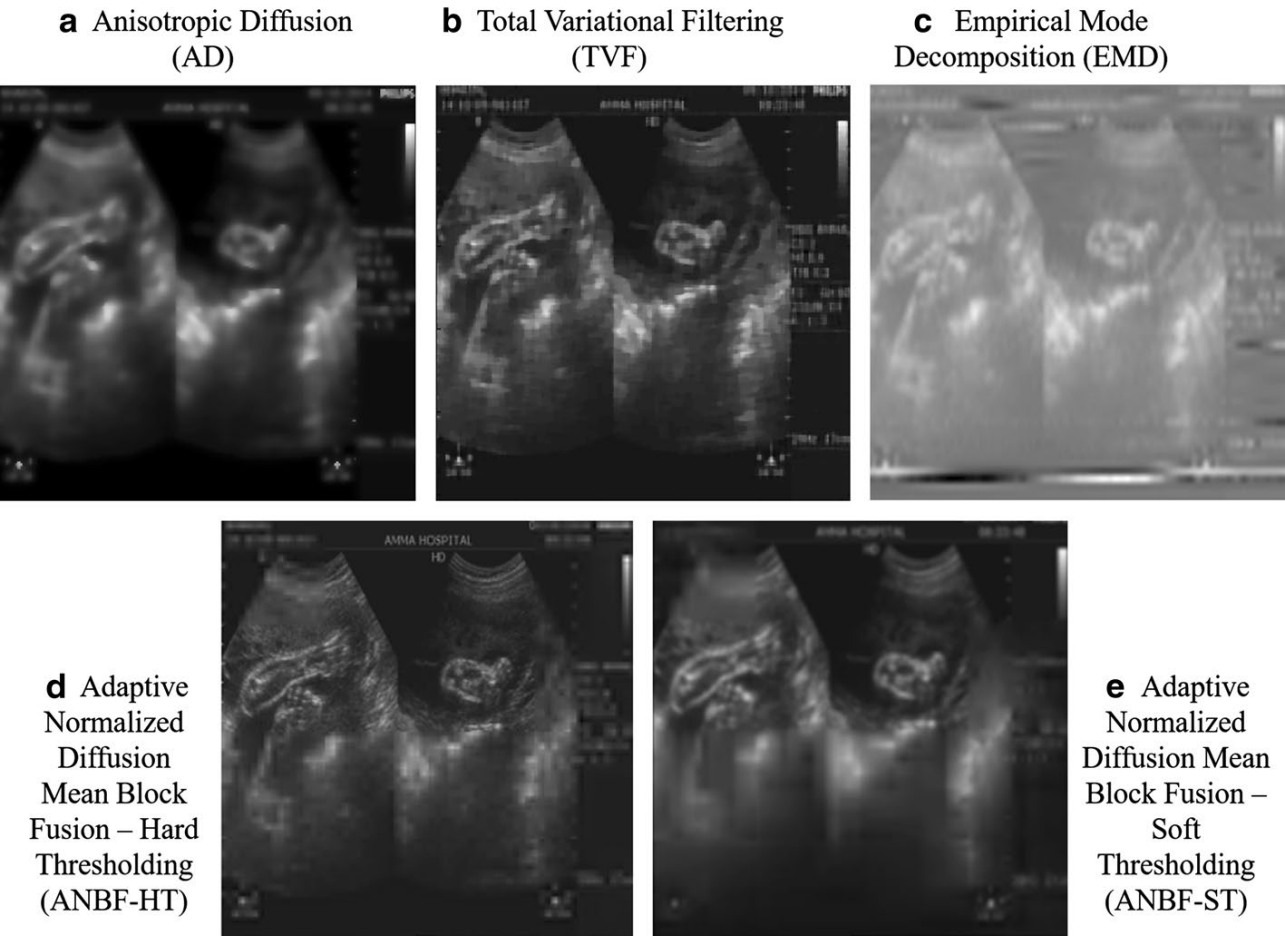
Test image from Fig. 2c denoised using **a** Anisotropic Diffusion with 44 iterations, **b** Total variational Filtering (TVF) with 65 iterations, **c** Empirical Mode Decomposition (EMD) with 5 Modes, **d** Adaptive Normalized Diffusion Mean Block Fusion-HT (ANBF-HT) with block size 32, **e** Adaptive Normalized Diffusion Mean Block Fusion-ST (ANBF-ST) with block size 16

Let us compare our proposed algorithm with these already proved techniques for denoising. The only drawback these methods face are from their dependence on gradient and number of iterations to reach the gradient image to preserve edges while denoising. Figures 9 and 10 are competitive images of the proved techniques AD, TVF and EMD with the proposed two fold techniques ANBF-HT with block size 16 and ANBF-ST with block size 8, for two test images in Fig. 2a, c.

From the visual perception of doctors at AMMA hospitals by seeing Figs. 9 and 10, they think our two fold proposed method clearly dominates the lot. The only case where they disagreed is on Fig. 9e which shows blocking artifacts due to lower block sizes. Higher block sizes avoid these artifacts. But the images are high in contrast to recognize objects in the image.

**Fig. 11:**
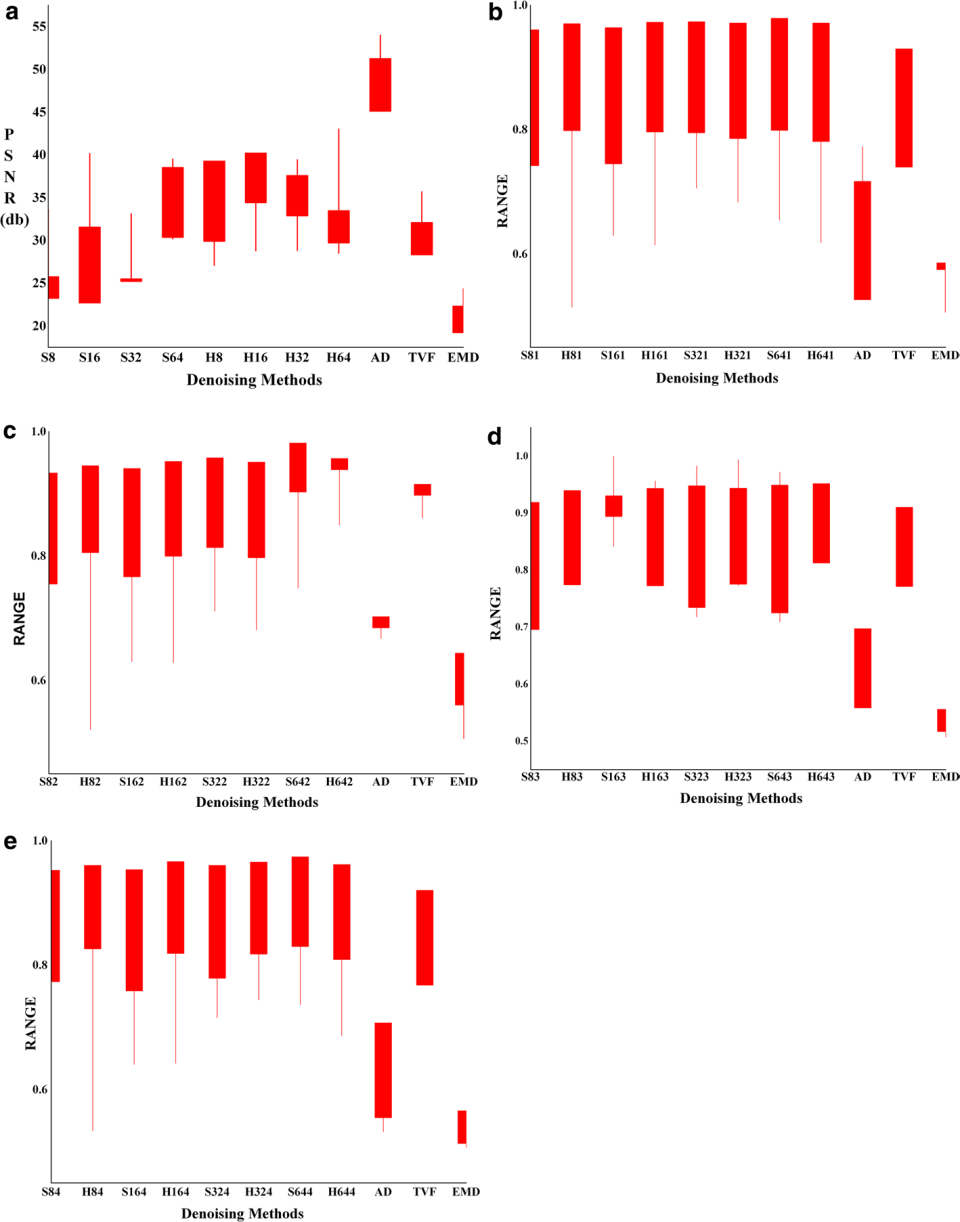
Comparative Quality metrics for various denoising algorithms **a** PSNR in db, **b** NCC, **c** ES, **d** IQI and **e** SSIM

The other three methods performed well to remove speckle but the quality of boundaries of objects in the images are poor, except for the TVF method. Checking for quality metrics to ascertain the superiority of denoising methods for US test images of Fig. 2. Calculating and plotting the metrics for the proposed methods (ANBF-HT and ANBF-ST with 4 block sizes each) against AD, TVF and EMD. Plot in Fig. 11 is a range plot showing the range of values on y-axis and the denoising methods on x-axis.

Figure 11a has PSNR distributions on the test images in Fig. 2 for proposed two fold techniques and the standard US denoising methods. The two fold methods lost it on PSNR compared to anisotropic diffusion (AD). Two fold techniques are showing better PSNR with respect to TVF and EMD. Figure 11b–e plots of NCC, ES, IQI and SSIM for individual test images. Close observations of the plots reveal the two fold techniques object boundary preservation compared to other models. Total variational filtering is the only method that protects object boundaries during denoising.

**Fig. 12:**
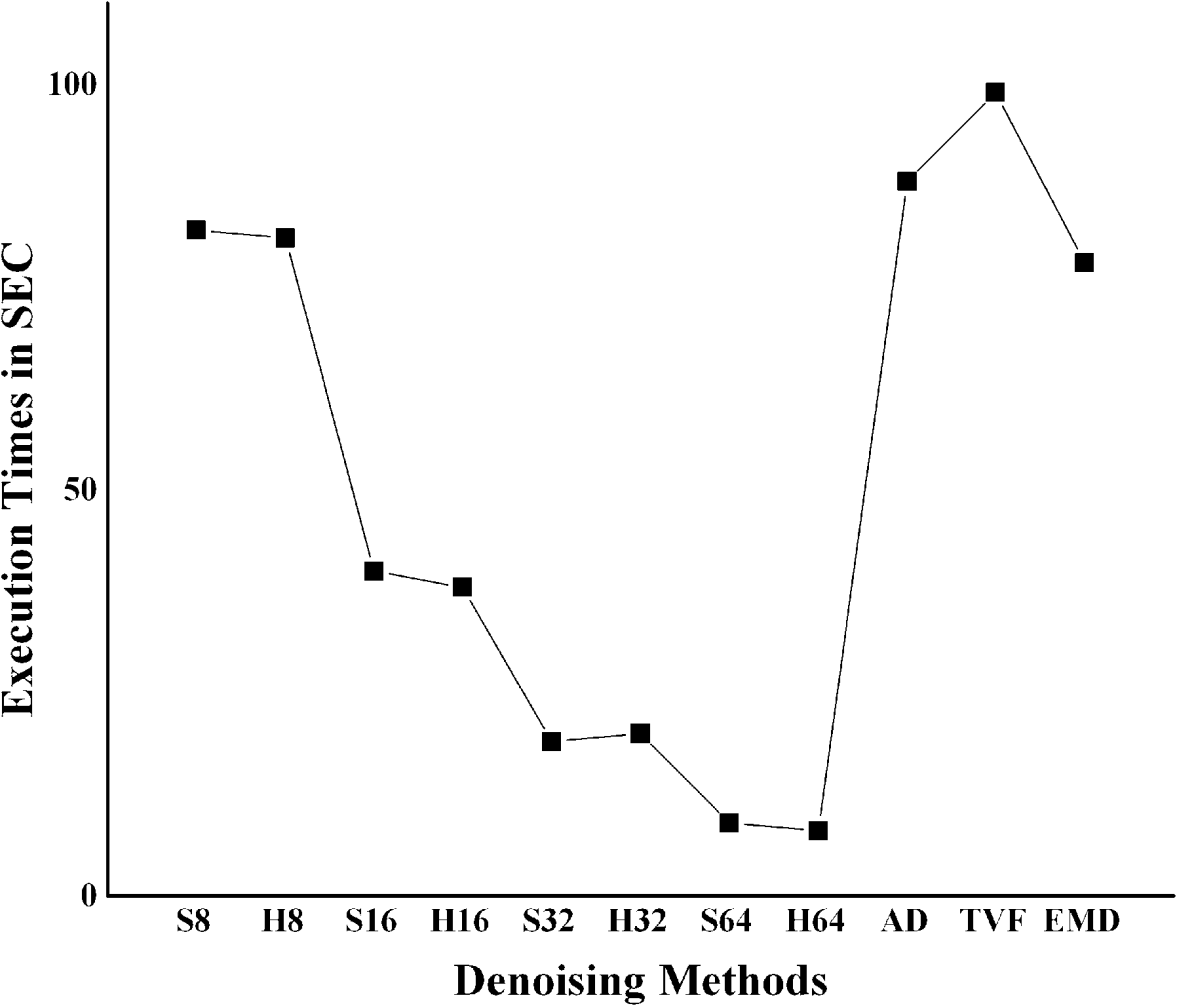
Execution times of denoising methods

The biggest drawback of AD, TVF and EMD is their iterative nature with in turn adds to execution time. MATLAB 13a is the programming language for achieving the goal. The machine is a HP laptop with i3 processor having a support RAM of 3GB. Finally comparisons on the execution time of each of the codes in MATLAB on the specified machine are given in Fig .12. These execution times are machine specific.

From Fig. 12, block size 8 based denoising methods with either HT or ST executes for 82 s. Block 16, block 32 and block 64 execute for an average of 40 sec, 20 sec and 9 sec respectively. AD and TVF are iterative gradient dependent methods and hence took 88 and 98 s for 40 iterations. Good denoised US images are generated by having a large number of iterations, which in turn slows the execution process. Same is the case with EMD.

## Conclusion

This paper proposes a twofold processing algorithm to reduce multiplicative speckle noise in ultrasound medical images for better visual quality. First fold reduces noise with wavelet block based thresholding, which affects image object boundaries and texture, thereby reducing the visual quality of objects in the image. The second fold restores object boundaries and texture from original ultrasound image through wavelet block fusion. Fusion rules and wavelet decomposition level selection between blocks of original US and threshold denoised US image is achieved using gradient histogram based Normalized Differential Mean (NDM) valve for adjacent blocks. Object blocks having boundary and texture are restored at lowest level from original US image and non-object regions from thresholded US image from the first fold. Hard and soft wavelet thresholding methods are incorporated in the first fold. The two fold methods are Adaptive Normalized Diffusion Mean Block Fusion - Hard Thresholding (ANBF-HT) and Adaptive Normalized Diffusion Mean Block Fusion - Soft Thresholding (ANBF-ST) for different block sizes. Four different block sizes are selected for testing such as 8, 16, 32 and 64 for both thresholding and fusion. The results are encouraging for clinical application, when compared to other popular methods. Quality metrics show a high degree of relativity with existing proven techniques for ultrasound image denoising such as anisotropic diffusion, total variational filtering and empirical mode decomposition.
